# Associations of history of vaccination and hospitalization due to infection with risk of monoclonal B-cell lymphocytosis

**DOI:** 10.1038/s41375-022-01514-3

**Published:** 2022-02-15

**Authors:** Nicholas J. Boddicker, Sara J. Achenbach, Sameer A. Parikh, Geffen Kleinstern, Esteban Braggio, Aaron D. Norman, Kari G. Rabe, Celine M. Vachon, Connie E. Lesnick, Timothy G. Call, Janet E. Olson, James R. Cerhan, Neil E. Kay, Curtis A. Hanson, Tait D. Shanafelt, Susan L. Slager

**Affiliations:** 1 Division of Computational Biology, Mayo Clinic, Rochester, MN USA; 2 Division of Clinical Trials and Biostatistics, Mayo Clinic, Rochester, MN USA; 3 Division of Hematology, Mayo Clinic, Rochester, MN USA; 4 School of Public Health, University of Haifa, Haifa, Israel; 5 Department of Hematology and Oncology, Mayo Clinic, Phoenix, AZ USA; 6 Division of Epidemiology, Mayo Clinic, Rochester, MN USA; 7 Department of Laboratory Medicine and Pathology, Mayo Clinic, Rochester, MN USA; 8 Department of Medicine, Division of Hematology, Stanford University, Stanford, CA USA

**Keywords:** Risk factors, Haematological diseases

## To the Editor

Monoclonal B-cell lymphocytosis (MBL) is an asymptomatic condition defined by the presence of circulating clonal B-cells in peripheral blood with a similar immunophenotype to that of chronic lymphocytic leukemia (CLL) without clinical symptoms or signs of disease [1]. MBL is a precursor to CLL [2, 3] and has been shown to be associated with risk of developing serious infection independent of progression to CLL [4, 5].

Prior studies have suggested that common infections (e.g., pneumonia, herpes zoster, sinusitis) are associated with subsequent increased risk of CLL [6, 7]. These studies infer that CLL patients may have a disturbed immune function prior to CLL diagnosis making the individuals more susceptible to infections. Infections may also have a role in the development of CLL, possibly through antigenic stimulation [8, 9]. Interestingly, individuals with MBL also have an increased risk of subsequent infections [4, 5], and some studies have suggested the clonal B-cell population may directly alter immune function [10]. Thus, it is currently unknown whether infection prior to developing CLL is due to infections being an inciting event to CLL, or if infections are strictly a consequence of having MBL.

Little is known about the history of infections and risk of developing MBL. In a study of 72 individuals with MBL and 380 controls, Casabonne et. al. reported increased risk of MBL in individuals with a history of pneumonia [11]. This study also reported that prior pneumococcal and influenza vaccinations were associated with reduced risk of MBL [11]. Moreover, two prior studies reported that select vaccinations may reduce risk of CLL [12, 13].

Here we evaluated prior history of serious infections and prior history of vaccinations with risk of MBL in a screening cohort of 1009 MBLs and 4419 known not to have MBL.

This study was approved by the institutional review boards of Mayo Clinic and Olmsted Medical Center, and participants provided written informed consent. Study participants were from the Mayo Clinic Biobank, a large-scale bio-repository of adult patients recruited through mailed invitation prior to their visit in primary care-based clinics, which ascertains patients’ vaccination history regardless of when vaccinations were given [14]. Participants had stored peripheral blood mononuclear cells (PBMC) collected from 7/14/2009 to 12/31/2020 that were screened for MBL, were residents of Olmsted county (location of Mayo Clinic), Minnesota at the time of sample collection, were 40 years of age or older with no prior history of hematologic malignancy, and had at least five years of medical history in the community prior to screening. For medical abstraction, we utilized the Rochester Epidemiology Project (REP), which is a population-based medical records-linkage system with access to the complete (in-patient and out-patient) medical records from all medical facilities in Olmsted county [15]. Using the REP, we queried medical records as far back as 1995 for any recorded history of vaccinations (regardless of when vaccination was given). Serious infections were defined as an individual who was hospitalized with an infection. Using the REP, we identified and reviewed all hospitalizations in the five years prior to sample collection for infections, following our published approach [4]. The five-year time period was selected to ensure the same amount of time of medical history. The medical record abstractor collecting data was blinded to MBL status.

Our MBL screening method has been previously published [5]. Briefly, PBMC’s were screened using an eight-color flow cytometry assay capable of detecting clonal B-cell events to the 0.005% level. Individuals with high-count MBL were those who had a percent clonal B-cell count ≥85% out of total B-cell count [3, 16].

Logistic regression was used to estimate odds ratios (OR) and 95% confidence intervals (CI) to evaluate the association of prior vaccinations and serious infections with MBL risk (overall and by low-count CLL-like), adjusting for age (continuous) at sample collection, sex, and race/ethnicity. *P* value was Bonferroni corrected for 30 tests (22 vaccines and 8 infection types) and *P* < 0.001 was considered statistically significant. Analyses were performed using SAS^®^ version 9.4 (SAS Institute, Cary, NC, USA) and R version 3.6.2.

A total of 5428 individuals were screened for MBL; 1009 (18.6%) were identified to have MBL and 4419 (81.4%) did not (controls) (Table 1). Among the MBL individuals, 866 (85.8%) had a CLL-like MBL subtype, and 947 (93.9%) were classified as low-count MBL. Individuals with MBL had a median age of 73.1 years compared to 65.8 years in controls (*p* < 0.001).

**Table 1:** Patient characteristics.

	Controls (*N* = 4419)	MBL (*N* = 1009)	Total (*N* = 5428)
Age years, median (range)	65.8 (40.2–101.3)	73.1 (41.0–97.5)	67.3 (40.2–101.3)
Age group			
40–49	470 (10.6%)	16 (1.6%)	486 (9.0%)
50–59	910 (20.6%)	113 (11.2%)	1023 (18.8%)
60–69	1352 (30.6%)	250 (24.8%)	1602 (29.5%)
70–79	1096 (24.8%)	373 (37.0%)	1469 (27.1%)
80+	591 (13.4%)	257 (25.5%)	848 (15.6%)
Sex (Male)	1531 (34.6%)	486 (48.2%)	2017 (37.2%)
Race/Ethnicity			
Non-Hispanic White	4231 (95.7%)	969 (96.0%)	5200 (95.8%)
Other	188 (4.3%)	40 (4.0%)	228 (4.2%)
MBL immunophenotype			
No MBL	4419 (100.0%)	—	4419 (81.4%)
CLL-like MBL	—	866 (85.8%)	866 (16.0%)
Atypical CLL-like MBL	—	36 (3.6%)	36 (0.7%)
Non-CLL like MBL	—	107 (10.6%)	107 (2.0%)
MBL Sub-classification			
Low-count MBL	—	947 (93.9%)	—
High-count MBL	—	62 (6.1%)	—

*MBL* monoclonal B-cell lymphocytosis, *CLL* chronic lymphocytic leukemia.

**Fig. 1:**
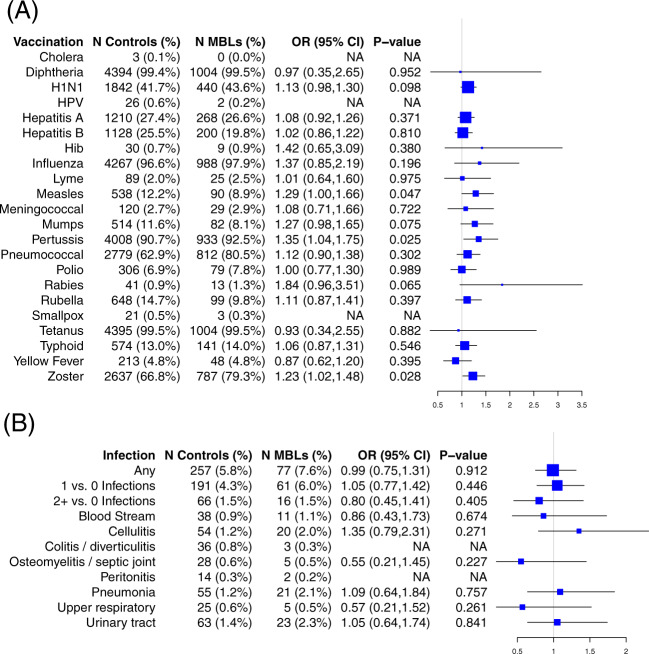
Prior history of vaccinations and serious infection wtih risk of monoclonal B-cell lymphocytosis (MBL). Association between monoclonal B-cell lymphocytosis (MBL) and history of vaccinations (**A**) and serious infections (**B**) prior to MBL screening, adjusting for age at sample collection, sex, and race/ethnicity. Zoster was restricted to individuals aged 50 and older. N number exposed, OR odds ratio, CI confidence interval, HPV human papillomavirus, Hib Haemophilus influenzae type B, NA not applicable (too few events (<5) for stable OR calculation).

Prior history of 22 types of vaccines were abstracted from medical records. The frequency of individuals that received the vaccines of interest ranged from 0.1% (e.g., cholera) to 99.5% (e.g., tetanus) (Fig. 1A). After adjusting for age at sample collection, sex, and race/ethnicity, none of the 22 vaccines were statistically significantly associated with MBL risk (ORs from 0.87 to 1.84) or low-count CLL-like MBL (ORs from 0.85 to 1.97, Supplementary Fig. 1A). Because the extent of medical record coverage varied by individuals, we also performed sensitivity analyses using the same time frame of medical record coverage for all individuals (5 years prior to MBL screening), and the results were consistent (data not shown). Finally, to account for varying introduction of vaccine immunization schedules, we stratified the cohort by age (younger than 65 years and 65+) and again the results were consistent across age groups (data not shown).

In the five years prior to sample collection, we abstracted eight classifications of infections associated with hospitalizations (Fig. 1B). A total of 257 (5.8%) controls and 77 (7.6%) MBL individuals had prior history of serious infection (Fig. 1B and Supplementary Table 1). After adjusting for age at sample collection, sex, and race/ethnicity, there was no association of any infection with risk of MBL (OR = 0.99; 95% CI: 0.75–1.31), or any specific infection (Fig. 1B). Additionally, there was no evidence of an association between the number of serious infections and risk of MBL (Fig. 1B). Similar results were found when we subset to low-count CLL-like MBL (Supplementary Fig. 1B).

This study involving >5000 individual (1009 MBL and 4419 controls) is the largest study to date to comprehensively investigate history of vaccination and serious infection prior to screening for MBL. We did not find any evidence of an association with either of these two immune agents. Our results conflict with a prior study that investigated history of vaccination and history of infection in 72 individuals with low-count MBL and 380 controls [11]. Specifically, that study reported individuals with low-count MBL were less likely to report having received pneumococcal and influenza vaccination and more likely to report having a history or pneumonia, meningitis, or influenza. These findings could be due to chance or due to the data collected from a self-reported questionnaire, whereas our data were abstracted from medical records.

Studies have documented that individuals with both low-count and high-count MBL are at increased risk of developing a subsequent serious infection, suggesting MBL is a marker of future infection risk [4, 5]. Individuals with low-count MBL have a 1.6- fold higher risk of hospitalization with infection [5], and individuals with high-count MBL have a 3.0- fold increased risk compared to controls [4]. We have also previously shown that individuals with early stage CLL are at 3.2-fold greater risk for subsequent infection than controls [4]. Based on our current study, prior history of serious infection appears to have no evidence of a role in the initiation of the MBL clone. Collectively, the current literature suggests that the greater the size of the clonal B-cell population, the greater the susceptibility to serious infections rather than infections causing development of the clone.

Moreover, our data may also lend some insight to prior reports of an association between history of infection and risk of CLL [6, 7], in that risk of CLL reported in these studies may be due to the CLL patients having preexisting MBL resulting in susceptibility to serious infections rather than history of serious infections resulting in CLL. Additional prospective studies are needed to better understand the relationship and temporality between MBL and infections with risk of CLL.

A limitation of our study is the cross-sectional design for MBL ascertainment which identified prevalent MBL cases, leading to the potential for prevalence-incidence bias and inability to address temporality. Accordingly, we were unable to evaluate the timing of vaccination/serious infection in relationship to MBL onset. Regardless, given the null results, this limitation is unlikely to impact our conclusions. Our definition of low-count or high-count MBL is based on the precent clonal B-cell counts out of the total B-cell counts. Although not the standard definition, this approach was first reported as a reliable way to distinguish high-count MBL from low-count MBL over a decade ago [16] and we have shown it has high sensitivity (92%) and specificity (97%) with the standard definition [3]. Last, we lacked the granularity to investigate risk of MBL based on type of infection (i.e., viral vs. bacterial), and we note that none of the individuals in this study were hospitalized due to Covid-19 infection prior to sample collection.

In summary, we found no evidence of an association of history of vaccinations or serious infections with MBL risk.

## Supplementary information

Supplemental Material
