# Supplementary material for: Associations of history of vaccination and hospitalization due to infection with risk of monoclonal B-cell lymphocytosis
Source: Leukemia. 2022 Feb 15;36(5):1404–7. doi: 10.1038/s41375-022-01514-3 (PMC8853183; doi:10.1038/s41375-022-01514-3)
Supplement: Supplementary file 1 — Supplemental Material [file 41375_2022_1514_MOESM1_ESM.docx]

Supplemental Table 1. Frequency of history of hospitalization with infection between low-count monoclonal B-cell lymphocytosis (MBL), high-count MBL, and controls

|  |  | Number of Events (%) | | |
| --- | --- | --- | --- | --- |
| Infection | **Controls**  **(N=4419)** | | **Low-Count MBL (N=947)** | **High-Count MBL (N=62)** |
|  | Infection during prior 5 years | | | |
| Any | 257 (5.8%) | | 70 (7.4%) | 7 (11.3%) |
| Number of infections |  | |  |  |
| 1 | 191 (4.3%) | | 57 (6.0%) | 4 (6.5%) |
| 2+ | 66 (1.5%) | | 13 (1.4%) | 3 (4.8%) |
| Blood stream | 38 (0.9%) | | 10 (1.1%) | 1 (1.6%) |
| Cellulitis | 54 (1.2%) | | 18 (1.9%) | 2 (3.2%) |
| Colitis/diverticulitis | 36 (0.8%) | | 3 (0.3%) | 0 (0.0%) |
| Osteomyelitis/septic joint | 28 (0.6%) | | 4 (0.4%) | 1 (1.6%) |
| Peritonitis | 14 (0.3%) | | 2 (0.2%) | 0 (0.0%) |
| Pneumonia | 55 (1.2%) | | 18 (1.9%) | 3 (4.8%) |
| Upper respiratory | 25 (0.6%) | | 5 (0.5%) | 0 (0.0%) |
| Urinary tract | 63 (1.4%) | | 20 (2.1%) | 3 (4.8%) |

A.

B.

Supplemental Figure 1. Association between low-count CLL-like monoclonal B-cell lymphocytosis (MBL) and history of vaccinations (A) and serious infections (B) prior to MBL screening, adjusting for age at sample collection, sex, and race/ethnicity. Zoster was restricted to individuals aged 50 and older. N: number exposed, OR: odds ratio, CI: confidence interval, HPV: human papillomavirus, Hib: Haemophilus influenzae type B, NA: not applicable (too few events (<5) for stable OR calculation).
